# Germline transformation of the stalk-eyed fly, *Teleopsis dalmanni*

**DOI:** 10.1186/1471-2199-11-86

**Published:** 2010-11-16

**Authors:** Ian A Warren, Kevin Fowler, Hazel Smith

**Affiliations:** 1 Department of Genetics, Evolution & Environment, University College London, Wolfson House, 4 Stephenson Way, London, NW1 2HE, UK; 2 Department of Entomology, Washington State University, Pullman, WA 99164-6382, USA

## Abstract

**Background:**

Stalk-eyed flies of the family Diopsidae have proven to be an excellent model organism for studying the evolution of ornamental sexual traits. In diopsid flies the eyes and antennae are borne at the end of lateral head projections called 'eye-stalks'. Eyespan, the distance between the eyes, and the degree of sexual dimorphism in eyespan vary considerably between species and several sexually dimorphic species show sexual selection through female mate preference for males with exaggerated eyespan. Relatively little is known about the molecular genetic basis of intra- or inter-species variation in eyespan, eye-stalk development or growth regulation in diopsids. Molecular approaches including comparative developmental analyses, EST screening and QTL mapping have identified potential candidate loci for eyespan regulation in the model species *Teleopsis dalmanni*. Functional analyses of these genes to confirm and fully characterise their roles in eye-stalk growth require the development of techniques such as germline transformation to manipulate gene activity *in vivo*.

**Results:**

We used *in vivo *excision assays to identify transposon vector systems with the activity required to mediate transgenesis in *T. dalmanni*. *Mariner *based vectors showed no detectable excision while both *Minos *and *piggyBac *were active in stalk-eyed fly embryos. Germline transformation with an overall efficiency of 4% was achieved using a *Minos *based vector and the *3xP3-EGFP *marker construct. Chromosomal insertion of constructs was confirmed by Southern blot analysis. Both autosomal and X-linked inserts were recovered. A homozygous stock, established from one of the X-linked inserts, has maintained stable expression for eight generations.

**Conclusions:**

We have performed stable germline transformation of a stalk-eyed fly, *T. dalmanni*. This is the first transgenic protocol to be developed in an insect species that exhibits an exaggerated male sexual trait. Transgenesis will enable the development of a range of techniques for analysing gene function in this species and so provide insight into the mechanisms underlying the development of a morphological trait subject to sexual selection. Our X-linked insertion line will permit the sex of live larvae to be determined. This will greatly facilitate the identification of genes which are differentially expressed during eye-stalk development in males and females.

## Background

In many species, sexual selection, the varying competitive success of individuals for access to mates [1,2], drives the evolution of exaggerated male displays or ornamental traits and female preference for such traits. The diopsid family of stalk-eyed flies exhibits a well documented and experimentally tractable example of an ornamental sexual trait [3-5]. Males and females have eyes laterally displaced from the head capsule on 'eye-stalks' [6] and the exaggeration of eye-stalks can be extreme with males having eyespans up to twice that of their body length [7,8]. Sexual dimorphism for eyespan, with males having much greater eyespan than females, has evolved several times within the Diopsidae [9]. Sexually dimorphic stalk-eyed fly species, such as *T. dalmanni *(formerly known as *Cyrtodiopsis dalmanni*; [10]), have become excellent model systems for the study of sexual selection due to the accumulation of evidence that exaggeration of male eyespan is driven by both female mate preference [7,8,11-14] and male-male competition [15,16].

Little is known about the genetic and developmental mechanisms that generate variation in sexual ornaments. Several approaches have been taken to identify potential candidate genes involved in eye-stalk development. Comparative gene expression studies have established that early development of the head capsule in diopsids is essentially similar to that of the non-hypercephalic *Drosophila melanogaster *[17,18]. QTL-based approaches have found micro-satellite polymorphisms correlated with increased eyespan [19]. Expression profiling using microarray chips made from expressed sequence tag (EST) libraries has identified a panel of genes associated with growth regulation, which are expressed in the developing head around the onset of metamorphosis [20]. These studies have demonstrated interesting correlations between gene expression patterns and morphology but functional assays are required to confirm causal relationships between candidate gene activity and eyespan. Further progress in understanding the genetic mechanisms underlying the exaggeration of male eyespan requires the development of techniques for manipulating the *in vivo *function and expression of candidate gene products.

Germline transformation and the insertion of foreign gene constructs into a genome are essential techniques for many manipulations of gene activity and function. The insertion of transgenes driven by stage and tissue specific promoters in single or binary systems allows the mis-expression or over-expression of any candidate gene, as well as the expression of altered forms of the gene product [21-23]. Transgenesis facilitates stage and tissue-specific RNAi-mediated gene knockdown [24-26]. In some species, germline transformation with appropriate constructs have been used to develop genome-wide enhancer trapping programmes to identify *cis*-regulatory elements and novel genes active in tissues of interest [27,28]. Genome-wide screens have also generated new mutations with relevant phenotypes via insertional mutagenesis [29,30].

Insect transgenesis is mediated by class II transposable elements. A construct containing the terminal inverted repeats of the transposon flanking the gene of interest is injected into embryos along with an exogenous source of transposase. This was first performed in *D. melanogaster *using the *P*-element [31]. Due to *P*-element activity being restricted to drosophilid species it was a further 10 years until a successful germline transformation was performed in a non-drosophilid insect, *Ceratitis capitata*, mediated by *Minos *[32,33]. The pan-specific activity of *Minos *and several other transposable elements, including *Mos1 *of the *Tc1/mariner *family [34], *Hermes *[35] and *piggyBac *[36-38] has enabled stable germline transformations in a wide range of insect and invertebrate species [39]. This was aided by the development of pan-specific promoters, such as *3xP3 *and *tetR*, combined with pan-specific reporters, such as EGFP and DsRed, which enable ready detection of transformation events regardless of the species [40-42].

Here we report the first stable germline transformation of the stalk-eyed fly, *T. dalmanni*. PCR-based excision assays were used to test the suitability of three potential transposable element vectors, *piggyBac, Mos1 *of the *Tc1/mariner *family and *Minos*. *piggyBac *and *Minos *showed activity in *T. dalmanni *embryos. Germline transformation was then achieved using *Minos *and the *3xP3-EGFP *construct. EGFP fluorescence was detected in the offspring of injected embryos and chromosomal insertion of the transgene was confirmed by Southern blot. In one line the insertion was stably inherited and X-linked while in others multiple copies of the insert were present, although inheritance of the transgenic phenotype did not follow the expected Mendelian model.

## Results

### Germline transformation of *T. dalmanni *using pMi[3xP3-EGFP]

**Figure 1:**
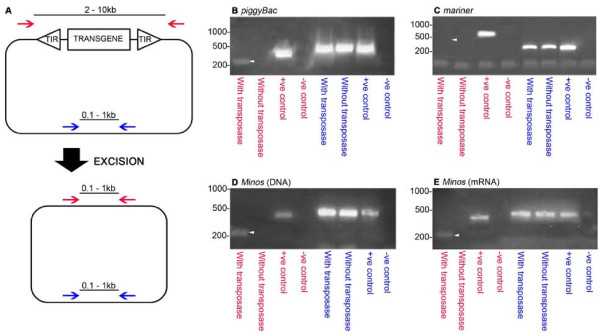
**PCR-based assays for transposon excision in *T. dalmanni *embryos**. (A) Schematic representation of a PCR-based excision assay. Donor plasmids contain the terminal inverted repeats (TIRs), from the *piggyBac, mariner *or *Minos *transposon, flanking a transgene. In the presence of transposase, donor plasmids undergo excision of the transgene. "Excision primers" (red arrows) flank the entire construct including the TIRs. PCR of the unexcised construct will give rise to a product too large to be amplified efficiently (2-10 kb) under standard conditions. Amplification post-excision produces a smaller product (0.1-1 kb). Excision primers are validated using modified donor plasmids, from which the element had been excised by digestion with an appropriate restriction enzyme, as control templates. "Extraction primers" (blue arrows) amplify part of the donor plasmid backbone to demonstrate successful extraction of the donor plasmid from injected embryos. PCR with these primers produces the same size product (0.1-1 kb) both pre-and post-excision. (B-E) Results of the excision assay using *piggyBac *(B), *mariner *(C) and *Minos *(D and E). Templates for PCR were: DNA extracted from embryos injected with donor plasmid and a source of transposase (with transposase); DNA extracted from embryos with donor plasmid without a source of transposase (without transposase); donor plasmid control templates (+ve control); water (-ve control). PCR reactions either used excision primers (red lettering) to test for excision of the transposable element, or extraction primers (blue lettering) to test for successful extraction of the plasmids from injected embryos (see Additional file 2: Table S1). White triangles denote the expected size of the excision primer PCR product post-excision of the element. For the *piggyBac *(B) and *mariner *(C) assays, a DNA source of transposase was used. For the *Minos *assays both a DNA source of transposase (D) and an mRNA source of transposase were tested (E). Excision was detected for *piggyBac *and both *Minos *assays when the donor plasmid was injected with a source of transposase but not in the *mariner *assay. No excision was detected when donor plasmids were injected without a source of transposase indicating a lack of endogenous transposase activity in the embryos. In all cases donor plasmids were successfully extracted from embryos.

Transposon vector systems can possess restricted host ranges [32,43]. PCR-based excision assays were used to assay the embryonic activities of three potentially suitable vectors, *piggyBac, mariner *and *Minos *in *T. dalmanni *(Figure 1). Excision was detected in the *piggyBac *and the *Minos *assays using both DNA and mRNA sources of transposase (Figure 1B, D &1E). There was no evidence of excision in the *Mos1/mariner *assay (Figure 1C).

We performed three rounds of micro-injections to investigate the possible use of *Minos *for the construction of transgenic stalk-eyed flies. *T. dalmanni *eggs were co-injected with a donor plasmid containing the sequence for *3xP3-EGFP *flanked by the *Minos *terminal inverted repeats (pMi[3xP3-EGFP]) and capped *Minos *transposase-encoding mRNA. Of 699 injected eggs, 74 (10.6%) produced viable adults (Table 1). Survivorship of uninjected eggs was significantly higher than of those injected with DNA/mRNA in all three rounds (*χ*^*2 *^= 23.421, d. f. = 1, *P *< 0.0001; *χ*^*2 *^= 20.346, d. f. = 1, *P *< 0.0001; *χ*^*2 *^= 41.684, d. f. = 1, *P *< 0.0001) but the proportion of eggs producing viable adults in these controls (40-60%) indicates that the fertility of egg-laying adults was low.

**Table 1:** Survivorship in each of three rounds of injections.

Experiment	Number of eggs (control)	% first instar larvae (control)	% pupae (control)	% adults (control)
1	135 (38)	6.7 (44.7)	4.4 (36.8)	4.4 (31.6)

2	123 (42)	13.0 (42.9)	7.3 (28.6)	4.1 (28.6)

3	441 (68)	32.1 (58.8)	15.9 (51.5)	14.3 (47.1)

Values are given for injected eggs with those for the corresponding controls (uninjected eggs) in brackets.

Transposition and integration of transgene DNA into the host germline was assayed by screening for EGFP fluorescence in the G1 progeny of outcrossed G0 (injected) individuals. In total, over 5000 G1 individuals were screened (Table 2). EGFP fluorescence was detected in offspring derived from two G0 individuals, labelled 17 and 34, giving an overall transformation efficiency of 4%. Fly 17 was a product of the first series of injections and fly 34 of the third series. Approximately 2% (3/126) screened offspring of fly 17 and 5% (15/283) of fly 34 were EGFP positive. Individual EGFP positive G1 offspring of both founders were outcrossed to wild type flies from the wild-type stock to generate lines of transgenic offspring for expression analysis and molecular characterisation of the insertions. Two lines were generated from founder 17 (17.1 and 17.2) and 11 from founder 34 (34.3-34.11, 34.14 & 34.15).

**Table 2:** Transformation frequencies for three injection experiments.

Experiment	Number of eggs injected	Number of G0 adults	Number of fertile G0 adults	Number of G1 individuals screened	Number of G0 transformants	Transformation efficiency (%)	Transformationrate (%)
1	135	6	2	323	1	50	0.74

2	123	5	3	34	0	0	0

3	441	63	45	4934	1	2.2	0.23

TOTAL	699	74	50	5291	2	4	0.28

Transformation efficiency is given as the percentage of fertile G0 adults giving rise to transgene-expressing G1 offspring (G0 transformants). Transformation rate is given as the percentage of injected eggs that gave rise to transformants.

**Figure 2:**
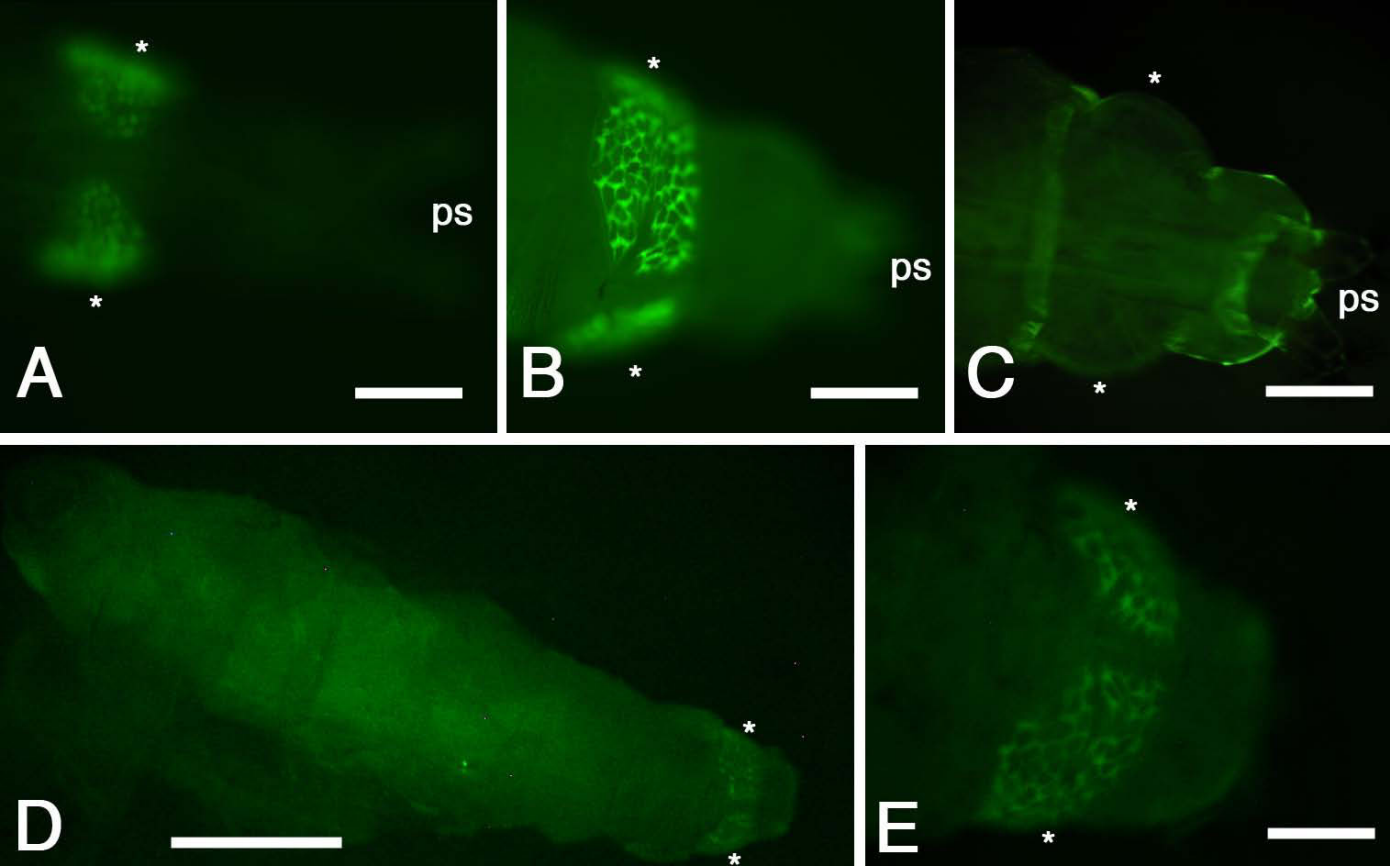
***EGFP *expression in transgenic larvae**. Larvae are from line 34.6 (A, B), line 17.1 (D,E) or non-transgenic (C). In both transgenic lines EGFP fluorescence was restricted to the posterior-most segments of the larva. Asterisks denote anal pads and **ps **denotes posterior spiracles. (A) Posterior end of a first instar larva showing *EGFP *expression in the anal pads. Note the position of the posterior spiracles. (B) Posterior end of a third instar larva showing strong EGFP fluorescence in mature anal pads. (C) Posterior end of a non-transgenic third instar larva. A degree of autofluorescence is visible in the cuticle but not in the anal pads. (D) Full length third instar larva showing *EGFP *expression at the posterior end. Autofluorescence is visible in the gut. (E) High power view of anal pad *EGFP *expression of the larva shown in (D). Scale bar in A = 100 μm; in B, C, and E = 200 μm; in D = 1 mm.

**Figure 3:**
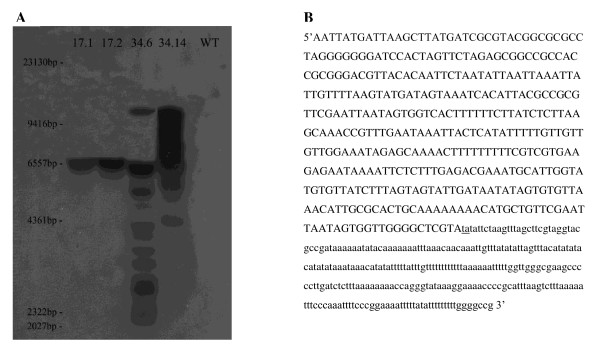
**Molecular characterisation of insertions**. (A). Southern analysis confirms transgene integration. DNA was extracted from individuals from four G1 individual derived lines carrying the transgene (17.1, 17.2, 34.6 & 34.14) and digested with *Eco*RI. The probe was generated from the *Not*I/*Sal*I fragment of pMi[3xP3-EGFP]. DNA from wild-type (WT) individuals was included as a control. For lines 17.1 and 17.2 single bands of the same size (approximately 6500 bp) are present. For lines 34.6 and 34.14 the multiple bands are indicative of multiple inserts at different locations in the genome. No bands are present in wild-type DNA. (B). Flanking sequence of right arm of an insert present in subline 34.6. The pMi[3xP3-EGFP] sequence is shown in upper case and the flanking genomic sequence in lower case. TA duplication, characteristic of a *Minos *mediated insertion event, is underlined.

In all *3xP3-EGFP *lines, *EGFP *expression was restricted to the larval anal pads (Figure 2). Expression was detected in all three larval instars. No expression was visible during embryonic stages of development and dissection of pupae and adult flies revealed no detectable fluorescence in the eyes or any other structure at either stage. Integration of the *Minos *element into the host genome was confirmed by Southern analysis (Figure 3A). A single 6500 bp fragment was detected in both lines of transgenic offspring of G0 founder 17 (17.1 and 17.2) indicating that a single insertion event had occurred in the parent fly. Multiple bands larger than the minimum expected fragment size (1339 bp) were detected in offspring of founder 34. The number and size of the bands was different in lines (34.6 and 34.14) derived from two different G1 individuals, suggesting multiple insertion events had occurred. Flanking sequence for one of the insertions was obtained by two-step gene walking [44]. The characteristic TA duplication associated with *Minos *mediated insertion was present (Figure 3B) but the flanking region showed no significant homology with any sequences in the stalk-eyed fly EST database [20] or any *D. melanogaster *sequences.

### Sex-linkage and stability of one transgenic insertion

*T. dalmanni *possess a chromosomal mechanism for sex determination [19] whereby females are the homogametic sex (XX) and males are the heterogametic sex (XY). X-linked insertions will therefore only be passed on to female offspring of a carrier male. G0 founder 17 produced two EGFP positive offspring both of which were female and were used to establish lines (17.1 & 17.2). To test for sex linkage of the insert, G2 crosses were set up for each G1-derived line (Table 3). Carrier males were individually crossed with virgin wild-type females and virgin carrier females were individually crossed with wild-type males. In both lines the segregation of the transgene with sex was consistent with the presence of a single X-linked insertion. For crosses involving a carrier male and a wild-type female, all male offspring were EGFP negative (from at least 24 individuals assessed in each line; Table 3). By contrast, for crosses involving a carrier female, EGFP positive progeny were equally distributed between the sexes (17.1: *χ*^*2 *^= 0.037, d. f. = 1, *P *= 0.85; 17.2: *χ*^*2 *^= 0.024, d. f. = 1, *P *= 0.89; Table 3). Stock populations were set up using *EGFP*-expressing (hemizygous) males and homozygous carrier females. Transgene expression was detected in all individuals sampled for eight consecutive generations, confirming that the insertion is stable.

**Table 3:** Sex linkage of the insert in transgenic line 17.

Line	Cross	G3 larvae		Sex of surviving G3 offspring			
		
		EGFP positive	EGFP negative	EGFP positive		EGFP negative	
				
				Male	Female	Male	Female
17.1	EGFP^+^♂ × wt ♀	98	111	0*	60	101	2

17.1	EGFP^+^♀ × wt ♂	81	79	28	26	29	30

17.2	EGFP^+^♂ × wt ♀	27	38	0*	24	19	3

17.2	EGFP^+^♀ × wt ♂	32	46	11	10	11	14

EGFP positive G2 individuals (EGFP^+^) were individually crossed with wild-type (wt) virgin flies of the opposite sex. Results are given as total output of five crosses. *Crosses involving EGFP^+ ^male flies, which did not produce any EGFP positive male offspring

### Multiple insertions and evidence of transgene suppression

Southern analysis indicated that multiple insertion events had occurred in G0 founder 34 and this was supported by segregation analysis suggesting independent insertions had occurred on the X and the autosomal chromosomes (Table 4). Founder 34 produced 11 G1 fertile, EGFP positive offspring, 6 of which were male. X-linkage of the insert was observed in the offspring of 4 of the G1 males, male EGFP positive G2 offspring being absent. For the other 2 G1 males, an autosomal pattern of inheritance was observed (Table 4). In almost all G1 crosses a significantly smaller proportion of offspring expressed the transgene than would be predicted by a strictly Mendelian model (*34.5*: *χ*^*2 *^= 9.8, d. f. = 1, *P *= 0.002; *34.7*: *χ*^*2 *^= 48.308, d. f. = 1, *P *< 0.0001; *34.8*: *χ*^*2 *^= 7.451, d. f. = 1, *P *= 0.006; *34.10*: *χ*^*2 *^= 65.627, d. f. = 1, *P *< 0.0001; *34.11*: *χ*^*2 *^= 37.692, d. f. = 1, *P *< 0.0001; *34.14*: *χ*^*2 *^= 45.534, d. f. = 1, *P *< 0.0001; *34.15*: *χ*^*2 *^= 20.547, d. f. = 1, *P *< 0.0001; Table 4).

**Table 4:** Segregation analysis for transgenic line 34.

Fly	Sex	G2 larvae		Sex of surviving G2 offspring			
		
		EGFP positive	EGFP negative	EGFP positive		EGFP negative	
				
				Male	Female	Male	Female
34.3	♀	12	20	3	3	1	2

34.4	♀	52	74	22	20	16	15

34.5	♂	12	33^§^	1	6	10	10

34.6	♂	50	71	13	17	22	16

34.7	♂	65	172^§^	0*	32	70	38

34.8	♀	24	47^§^	7	13	8	15

34.9	♀	7	11	5	2	1	2

34.10	♀	15	103^§^	4	6	26	35

34.11	♂	30	100^§^	0*	14	27	6

34.14	♂	96	215^§^	0*	72	41	17

34.15	♂	10	43^§^	0*	5	5	6

Surviving fertile EGFP positive G1 progeny of the G0 individual, fly 34, were individually crossed with wild-type virgin flies of the opposite sex to generate G2 offspring. G2 offspring were screened for EGFP fluorescence during the third larval instar and sex was recorded when individuals eclosed. Not all G2 progeny were reared to adulthood. A single X-linked insertion in a male G1 carrier would lead to all female offspring being EGFP positive and all males being EGFP negative. Autosomal insertions would be inherited by both female and male G2 offspring. *In four male derived sublines there was a significant sex bias among EGFP positive offspring with EGFP positive males being absent. Both male and and female EGFP positive offspring were produced by males 34.5 and 34.6.

§There was a significant deficit of EGFP positive offspring in seven sublines.

Previous studies which have observed non-Mendelian transmission of multiple copy transgenes have shown that the reduced recovery of the transgenic phenotype was linked to transgene instability [45]. To assess whether a similar mechanism might account for the skewed recovery of EGFP positive offspring, we set up a series of test crosses. Five EGFP negative G2 females which would have inherited the transgene carrying X chromosome from their male parent (34.14) were individually crossed with wild-type males. All EGFP negative females produced EGFP positive G3 offspring, although the proportion of EGFP positive offspring (15/141 pooled over 5 families) was significantly below 50% (*χ*^*2 *^= 60.8, d. f. = 1, *P *< 0.001). This indicates that the transgene was still present in the EGFP negative female parents but its expression had been suppressed by a subsequently reversible mechanism.

## Discussion

In this paper we describe the stable germline transformation of a stalk-eyed fly, *T. dalmanni*. Previously, four transposon vectors have been used successfully in non-drosophilid insect transgenesis. We employed an *in vivo *excision assay to test for transposon activity in stalk-eyed fly embryos. No activity of the *Mos1 mariner *transposon was detected. Transformation efficiencies obtained with *mariner *based vectors are generally lower than those seen with *Minos *or *piggyBac *[30,34,40,46]. However, members of the *mariner *family have been detected in the several diopsid species, including *T. dalmanni *[47]. *Mos 1 *transposon repression in this species could be due to self-inhibition of *mariner *activity by endogenous elements [48]. Both *piggyBac *and *Minos *transposons showed activity in *T. dalmanni *embryos. We used *Minos *in conjunction with a *3xP3-EGFP *marker construct for germline transformation.

The observed transformation rate of 4% among injected individuals is comparable to those commonly reported in insect transformation systems [38,49,50] but the overall efficiency of the protocol was reduced by the low proportion of injected eggs giving rise to viable adults. In part this was probably due to trauma associated with the injection procedure but low fertility was also a factor. Low fertilities have been documented for other laboratory and field populations of *T. dalmanni *and appear to be common among diopsids [51]. Low survivorship and efficiencies have been reported in transformation protocols for other insect species, especially in initial transformations experiments, but these have not prevented further use of the protocol [33,35,52,53].

The *3xP3-EGFP *construct was selected because its activity is pan-species specific and it has proved simple to detect in live larvae and adults. Given that *3xP3 *is based on the *Pax6 *promoter it would be expected to drive *EGFP *expression in the larval and adult eye and brain [54]. However, studies of Dipterans have frequently reported expression in the anal pads or papillae [49,54,55]. Here, in all *T. dalmanni *transformant lines, expression was restricted to the larval anal pads but not detected in the eyes or brains of larvae or adults. A similar pattern of larval expression was reported for *3xP3-EGFP *transgenic *Musca *[49], indicating that the eye/brain expression driven by this promoter is susceptible to position effect-based suppression [56]. The lack of adult eye expression in *T. dalmanni *is somewhat unusual. However previous Dipteran studies used eye colour mutants (in which adult eye fluorescence is visible without dissection) to identify transgenic founders and so would not have been able to recover insertions with purely larval expression. Such eye colour mutants are not available in *T. dalmanni.*

In lines carrying multiple inserts, expression of the transgenic phenotype was detected in fewer than expected offspring. EGFP negative individuals were able to produce EGFP positive offspring, suggesting that, in this case, loss of expression was not solely due to transgene loss but, at least in part, to suppression of transgene activity [45]. A variety of transcriptional and post-transcriptional mechanisms that lead to transgene suppression have been described in the literature [57,58]. Position effects and position effect variegation are also commonly observed in insects [56,59]. Further experiments would be required to determine which, if any, of these mechanisms are in operation here.

In the lines established from one founder (17) a single *3xP3-EGFP *insertion was stable and X-linked. Crosses between males hemizygous for this insert with wild-type females will result in all female, but no male, offspring expressing the transgene. This allows live male and female larvae to be distinguished unlike previous methods for sexing diopsid larvae which required the sacrifice of the individual [60]. From an evolutionary standpoint there is considerable interest in identifying and characterising the timing and level of expression of the genes on which selection acts to regulate eyespan in sexually dimorphic diopsid species such as *T. dalmanni*. One potentially powerful approach is to compare gene expression during the development and growth of the eye-stalks in males and females. The transgenic method enables groups of living male and female larvae to be independently cultured and manipulated. Differential gene expression and development of the sexes can be assessed throughout larval and pupal development using microarrays or other high throughput approaches [61].

The availability of an effective transgenic protocol enables multiple experimental approaches to be developed in stalk-eyed flies. These include over-expressing or knocking down candidate genes for developmental or other functions, morphological analysis and sperm competition assays [62,63]. EST sequencing and microarray analysis of *T. dalmanni *eye-antennal imaginal discs has identified a number of candidate genes for effects on eye-stalk development including the cell cycle progression regulators, *crooked leg *(*crol*) and *cdc2 *[20]. Our establishment of a successful transgenic protocol makes functional analysis of such genes possible. Several genes have been found for which variation in glutamine repeats is correlated with variation in eyespan including *corto, tousled-like kinase *(*tlk*), and *ecdysone-induced protein 75B *(*Eip75B*) [64]. Transgenics could be used to manipulate the number of glutamine repeats in order to study the effects on associated morphological traits [65].

Transgenic approaches would further benefit from the development of binary GAL4-UAS systems for targeted gene expression and to facilitate the analysis of constructs that would result in lethality or infertility [22]. Such systems are well established in *D. melanogaster *and have recently become available in non-model systems, such as *Bombyx mori *[26]. The constructs developed for non-drosophilids are *piggyBac*-based but for some applications would benefit from having a second vector such as *Minos *to supply a stable transgenic source of *piggyBac *transposase. In this context, it is advantageous that we have shown that both *Minos *and *piggyBac *are active in *T. dalmanni.*

## Conclusions

In this paper we describe the first transgenic protocol to be developed in *T. dalmanni*, a species that exhibits an ornamental sexual trait. Stable germline transformation technology will facilitate a variety of experimental approaches with the potential to greatly enhance understanding of sexual selection in general and the evolution and development of exaggerated sexual traits in particular. The X-linked *EGFP*-expressing insertion we have described allows live larval sexing. This can be combined with existing microarray-based methods for analysing differential gene expression in *T. dalmanni *[20] in order to identify sex-specific candidate genes at key stages of larval and pupal development in diopsids.

## Methods

### Stock population, experimental flies and egg collection

A laboratory-adapted population of *T. dalmanni *founded from flies collected from Ulu Gombak, Peninsular Malaysia in 1993 was used for these experiments. The population has been maintained in cage culture at 25°C, in a 12 h:12 h light:dark light cycle regime and fed pureed sweetcorn twice weekly. In order to minimise inbreeding, population size has been kept high (> 200 individuals). Eggs for experiments were obtained from laying populations of sexually mature flies (> 500 flies in groups, each consisting of 9-15 flies at a ratio of 2:1 females:males), housed in inverted 1.5 L plastic containers with bases of damp cotton wool and filter paper. Bases were lined with dark blue paper discs, on which freshly laid eggs could be readily identified. Eggs were collected from all groups after an oviposition period of 1.5 h and pooled on moistened paper towel discs for ease of subsequent handling.

### Microinjections

A protocol for embryonic injections [66] based on *D. melanogaster *protocols was used. Groups of 10-30 embryos with intact chorions were arranged side by side on a coverslip attached to a slide, left for 5 minutes to desiccate and adhere to the coverslip, then covered with halocarbon oil 700 and placed on an inverted microscope (IDOC, Zeiss, Ukraine). Microinjections were performed using a needle attached to a micro-manipulator (MN-153, Narishige, Japan), an electric microinjector (IM-30, Narishige, Japan) and an oil air compressor (Jun-air, Norgren, Denmark). Microinjection needles [67] were made from borosilicate glass capillaries (length 100 mm, outer diameter 1.0 mm, inner diameter 0.58 mm) and shaped using a P-97 needle puller (INTRACEL, Sutter Instruments, UK).

Needles were back-filled by capillary action. Injection buffer was passed through a 2 μm Acrodisc syringe filter (Pall Corporation, UK) to avoid dust blocking needles. Prior to use, the injection mix was centrifuged on a bench top centrifuge for 15-30 minutes at 14,000 rpm. Embryos aged between 0.5 and 3 hours post laying (before pole cell formation) were injected in the posterior end (Additional file 1: Figure S1).

### Excision assays

The donor plasmids were: *piggyBac *- pBac[3xP3-EGFPafm] [40], *mariner *- pMos[3xP3-EGFPafm] [41], *Minos - *pMiLRTetR(L) [68]. The helper plasmids were: *piggyBac - *phsp-pBac [69], *mariner *- pKhsp82MOS [70] and *Minos - *pHSS6hsILmi20 [68]. *Minos *transposase mRNA was synthesised using the MEGAscript^® ^T7 Kit (Ambion Inc., USA) and pBlueSKMimRNA linearised with *Not*I as the template [71].

For the *piggyBac *and *mariner *excision assays, 50-100 eggs were injected with 250 μg/ml of both helper and donor plasmid in injection buffer (150 μM NaH_2_PO_4_, 850 μM Na_2_HPO_4_, 5 mM KCl at pH7.6-7.8, 0.05% phenol red). For the *Minos *assays, embryos were injected with 300 μg/ml of donor plasmid and 150 μg/ml helper plasmid [68] or 500 μg/ml donor plasmid and 300 μg/ml transposase mRNA [71]. Following a 48 hour incubation period DNA was extracted and PCR reactions performed [68]. Primers designed to detect excision and successful plasmid extraction from the injected embryos are listed in Additional file 2: Table S1).

Excision primers were tested using modified donor plasmids as control templates. In each control template a fragment of the sequence between the two primer sites had been excised by digestion with an appropriate restriction enzyme to produce a shorter and reliably amplifiable product under standard PCR conditions. Restriction enzymes were: *Eco*RV with pBac[3xP3-EGFPafm]; *Hind*III with pMos[3xP3-EGFPafm]; and *Not*I with pMiLRTetR(L).

### Transformation of *T. dalmanni*

Eggs were injected with 500 μg/ml donor plasmid (pMi[3xP3-EGFP] [71]), and 300 μg/ml *Minos *transposase mRNA in injection buffer (150 μM NaH_2_PO_4_, 850 μM Na_2_HPO_4_, 5 mM KCl at pH7.6-7.8, 0.05% phenol red). Following injection, halocarbon oil was removed and the coverslip containing the embryos transferred to pureed sweetcorn in a 90 mm Petri dish lined with moist cotton wool/filter paper to hatch and complete larval development at 25°C. Upon pupation (after approximately 10 days), individuals were transferred to lined 1.5 L plastic containers. As a control for the effect of microinjection, a group of uninjected embryos was transferred directly to sweetcorn. Survivorship of injected and control embryos was recorded at hatching, pupariation and eclosion.

Sexually mature adults derived from injected (G0) individuals were crossed with 1-3 wild-type virgin individuals of the opposite sex to generate offspring (G1). All eggs produced were collected and transferred to pureed sweetcorn in Petri dishes lined with moist cotton wool for further culture and surviving larvae screened at third instar stage. An average of 105.8 larvae per G0 founder were checked for EGFP fluorescence using a UV-dissecting microscope (Leica MZII, Leica, Germany) and photographed using a Nikon 5100 digital camera.

### Genetic characterisation, breeding and maintenance of insertions

All surviving G1 individuals which had shown EGFP fluorescence as larvae were crossed with 1-3 virgin wild-type individuals of the opposite sex. To characterise and test for stability of any insertion events, the offspring were screened for EGFP fluorescence as larvae and their sex as adults recorded subsequently. The expression pattern in each lineage was assessed at embryonic and all three larval stages. Dissections of pupal and adult heads were also carried out to test for eye/brain expression. For X-linked lines, ten pairwise crosses were set up between *EGFP*-expressing males and females to create homozygous/hemizygous stocks. 25 offspring of each cross were screened for *EGFP *expression. Pairs producing 100% *EGFP*-expressing offspring were used to set up a stock population.

### Southern analysis

For the extraction of good quality genomic DNA, the head, wings and abdomen were removed from 20-30 flies. The remaining tissue was flash frozen in liquid nitrogen for 1 minute, then homogenized using a flame-blunted pipette tip in 300 μl HM buffer (0.1 M NaCl, 0.2 M Sucrose, 0.1 M tris-HCl pH9.1, 0.05 M EDTA, 0.5% SDS, 0.33 mg/ml proteinase K). Following 2-3 hours incubation at 55°C, 85 μl of 5 M NaCl was gently added and the mixture left at 4°C for 20 minutes prior to centrifugation at 14000 g for 20 minutes at 4°C. The supernatant was placed in a fresh eppendorf tube, 1 ml of cold EtOH was added and the DNA precipitate at the interface spooled onto a flame-modified glass pipette. Spooled DNA was washed in 70% EtOH and dissolved in 30 μl TE buffer. Approximately 10 μg genomic DNA was digested with *Eco*RI, size-separated by agarose gel electrophoresis and blotted onto nylon membranes (Amersham Biosciences, UK). A 762 bp *Sal*I/*No*tI fragment of pMi[3xP3-EGFP] was used as a probe. Radioactive labelling, hybridisation and visualisation were carried out using standard techniques [72].

### Two-step gene walking

DNA extraction from individual female transgenic flies was performed [60]. PCR and sequencing primer details are listed in Additional file 3:Table S2. 50-500 ng genomic DNA was used as the template for the two-step PCR reactions [44] with reaction conditions: 94°C for 10 minutes; 30 cycles of 30 seconds at 94°C, 30 seconds at 56°C and 3 minutes of 72°C; 1 cycle of 30 seconds at 94°C, 30 seconds at 40°C, 3 minutes at 72°C; 30 cycles of 30 seconds at 94°C, 30 seconds at 56°C, 3 minutes at 72°C; and a final stage of 10 minutes at 72°C. PCR products were analysed by agarose gel electrophoresis, DNA was isolated from those reactions that gave products (PCR purification kit, Qiagen, UK) and sequenced by Macrogen (Korea).

## Authors' contributions

IAW, KF and HS jointly conceived the study, participated in the design and coordination, and drafted the manuscript. IAW performed the experiments and carried out the analyses. All authors have read and approved the final manuscript.

## Supplementary Material

Additional file 1 **Figure S1: Early development and external morphology of stalk-eyed fly embryos**. Figure showing pole cell formation and the distinct morphology of the anterior and posterior poles in embryos with their chorions attached. Click here for file

Additional file 2 **Table S1: Function, sequence and expected product sizes for primers used in excision assays**. Table showing primers designed to test for excision of the transposon or successful extraction of the donor plasmid from the injected embryo. Click here for file

Additional file 3 **Table S2: Primers used for two-step gene walking**. Table showing PCR and sequencing primers used. Click here for file
