# Supplementary material for: Germline transformation of the stalk-eyed fly, Teleopsis dalmanni
Source: BMC Mol Biol. 2010 Nov 16;11:86. doi: 10.1186/1471-2199-11-86 (PMC2999598; doi:10.1186/1471-2199-11-86)
Supplement: Additional file 1 — Figure S1: Early development and external morphology of stalk-eyed fly embryos. Figure showing pole cell formation and the distinct morphology of the anterior and posterior poles in embryos with their chorions attached. [file 1471-2199-11-86-S1.PDF]

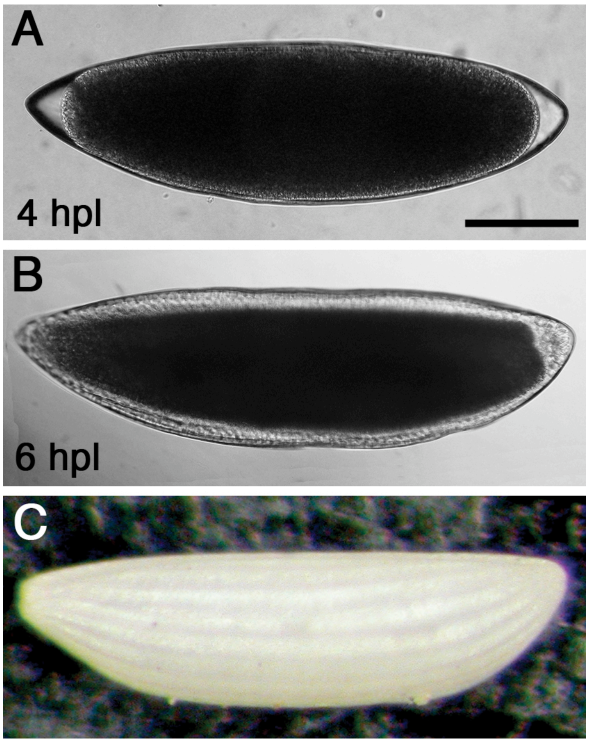


**Additional file 1.** Early development and external morphology of stalk-eyed fly embryos**.** In all panels, posterior is to the right. (A) and (B). Light microscope photographs of embryos of *T. dalmanni* at 4 and 6 hours post laying (hpl), respectively. Pole cells cannot be detected at 4hpl but are evident from 6 hpl. (C). Light microscope photograph of *T. dalmanni* embryo with chorion attached to demonstrate the distinct morphology of the anterior and posterior poles. A more severe taper is seen at the posterior pole, which is pointed while the anterior pole appears blunt. Scale bar 75μm.
