# Supplementary material for: Germline transformation of the stalk-eyed fly, Teleopsis dalmanni
Source: BMC Mol Biol. 2010 Nov 16;11:86. doi: 10.1186/1471-2199-11-86 (PMC2999598; doi:10.1186/1471-2199-11-86)
Supplement: Additional file 2 — Table S1: Function, sequence and expected product sizes for primers used in excision assays. Table showing primers designed to test for excision of the transposon or successful extraction of the donor plasmid from the injected embryo. [file 1471-2199-11-86-S2.PDF]

Assay	*Test*	*Primer Name*	*Sequence (5'-3')*	*Expected band size with excised donor plasmid (bp)*	*Expected band size with control plasmid (bp)*
piggyBac	Excision	PbacRev1<br>PbacFor1	GGTCGAGTAAAGCGCAAATC<br>TACGCACCATATACGCATCG	269	361
	Plasmid extraction	PbacConf1<br>PbacConr1	AGGAGAAAATACCGCATCAG<br>CACAGATTTAAAGAACCAAAA	450	450
mariner	Excision	MosRev1<br>MosFor1	GCGTAAGAACGGGGACCTA<br>ATTAGGCACCCCAGGCTTTA	402	510
	Plasmid extraction	MosConf1<br>MosConr1	AACGATCAAGGCGAGTTACA<br>GTATTGACGCCGGGCAAG	246	246
Minos	Excision	MiR-hydei<br>MiL-Lorist	TGCATTCTCTATGCT<br>CCAGCTGGCTTATCGAAA	211	442
	Plasmid extraction	Lorist6conl1<br>Lorist6conr1	GGCTGAACACACCGTTGAT<br>GGGACAGCAGAAGACCTGAC	465	465

Additional file 2. Function, sequence and expected product sizes for primers used in excision assays. Primers were designed to either test for excision of the transposon or for successful extraction of the donor plasmid from the injected embryo. MiR-hydei and MiL-Lorist were designed by Klinakis *et al.* (2000).
