# Supplementary material for: Germline transformation of the stalk-eyed fly, Teleopsis dalmanni
Source: BMC Mol Biol. 2010 Nov 16;11:86. doi: 10.1186/1471-2199-11-86 (PMC2999598; doi:10.1186/1471-2199-11-86)
Supplement: Additional file 3 — Table S2: Primers used for two-step gene walking. Table showing PCR and sequencing primers used. [file 1471-2199-11-86-S3.PDF]

Primer name	***Sequence (5'-3')***	***Position on pMi[3xP3 -EGFP]***	***Position on Insert***
2stepfor_A	CTG CAT TCT AGT TGT GGT TTG TCC	1698-1721	Right arm
2stepfor_B	GTA TGA TAG TAA ATC ACA TTA CG CC G	1852-1877	Right arm
2stepfor_C	CAC ACC TCC CCC TGA ACC	1570-1587	Right arm
2steprev_A	ATC AAG CTT ATC GAT ACC GTC G	477-498	Left arm
2steprev_B	GAA CTT CAG GGT CAG CTT GC	899-918	Left arm
2steprev_C	AGC TTA TCG ATA CCG TCG ACC TC	472-494	Left arm
2step_seq_g	GCA TTC TAG TTG TGG TTT GTC C	1700-1721	Right arm

Additional file 3. Primers used for two-step gene walking and product sequencing. Primers 2stepfor _A, 2stepfor _B, 2stepfor _C, 2steprev _A, 2steprev _B, and 2steprev _C were used in two-step PCR reactions. Primers 2stepfor_A and 2step_seq_g were used for sequencing. Position refers to location on the original donor plasmid, pMi[3xP3-EGFP]. The 2.025kb insert runs from position 100 to 2125 on the plasmid.
